# Supplementary material for: Detection and Characterization of an H9N2 Influenza A Virus in the Egyptian Rousette Bat in Limpopo, South Africa
Source: Viruses. 2023 Feb 10;15(2):498. doi: 10.3390/v15020498 (PMC9958621; doi:10.3390/v15020498)
Supplement: Supplementary file 1 [file viruses-15-00498-s001.zip › viruses-2187636-supplementary.pdf]

viruses

MDPI

Supplementary material

Detection and Characterization of the an H9N2 Influenza A Virus in the Egyptian Rousette Bat in Limpopo, South Africa

Rochelle Rademan, Marike Geldenhuys and Wanda Markotter

Supplementary methods

18S rRNA internal control PCR assay

The primers that were used for the internal control PCR assay included the 18S rRNA FW-forward primer (5'- CGC CGC TAG AGG TGA AAT TC- '3) and the 18S rRNA RV-reverse primer (5′-CGA ACC TCC GAC TTT CGT TCT- '3) from Nakahata et al., 2006 that target a 100 bp amplicon within the 18S rRNA. The 50 µl reaction mixture for the internal PCR assay consisted of 2 µl randomly primed cDNA template, 1x DreamTaq buffer (Thermo Fisher Scientific, USA), 0.2 µM 18S rRNA FW-forward and 18S rRNA RV-reverse primer (Integrated DNA Technologies, USA), 0.2 mM dNTP mixture (Thermo Fisher Scientific, USA), 3.0 mM $MgCl_2$, 1.25 U DreamTaq Polymerase (Thermo Fisher Scientific, USA), and 37.75 µl nuclease-free water (Ambion, Thermo Fisher Scientific, USA). The PCR assay cycling conditions include an initial cycle of 95°C for 1 minute, 40 cycles of denaturation at 95°C for 30 seconds, annealing at 55°C for 30 seconds, and extension at 72°C for 1 minute, followed by a final extension cycle of 72°C for 10 minutes.

Supplementary Tables and Figures

Table S1: Influenza A virus segment-specific primers (reference genome: H9N2, accession: MH376902-09) for the full genome amplification and sequence analysis of the positive sample.

**Segment**		**5′-Primers-3′**	**Size (b)**
**PB2**	Forward	ATC **AGC GAA AGC AGG** TCA AAT ATA TTC	27
	Middle	GCT GTT GTA TCT GCT GAT CCA CTA GC	26
	Reverse	T ATC **AGT AGA AAC AAG G** TCG TTT TTA AAC	29
**PB1**	Forward	ATC **AGC GAA AGC AGG** CAA ACC ATT TG	26
	Middle	CAG ATG CTA GTT GGA TTG AAC	21
	Reverse	T ATC **AGT AGA AAC AAG G** CAT TTT TTC	26
**PA**	Forward	ATC **AGC GAA AGC AGG** TAC TGA TCC	25
	Middle	GAC CTG CAA CTG CTC AAA TCG C	22
	Reverse	TC **AGT AGA AAC AAG G** TAC TTT TTT GG	26
**NP**	Forward	ATC **AGC AAA AGC AGG** GTA GAT AAT CAC TC	29
	Reverse	T ATC **AGT AGA AAC AAG G** GTA TTT TTC	27
**NS**	Forward	ATC **AGC AAA AGC AGG** GTG ACA AAA AC	26
	Reverse	AT ATC **AGT AGA AAC AAG G**GT G	21
**M**	Forward	ATC **AGC AAA AGC AGG** TAG ATR TTK AAA G	28
	Reverse	ATC **AGT AGA AAC AAG G** TAG TTT TTT ACT CC	30
**HA**	Forward	ATC **AGC AAA AGC AGG** GGA ATT TCT TAA C	28
	Reverse	T ATC **AGT AGA AAC AAG G** GTG TTT TTG	26
**NA**	Forward	ATC **AGC AAA AGC AGG** AGT GAA A ATG AAT CC	30
	Reverse	T ATC **AGT AGA AAC AAG G** AGT TTT TTC	26

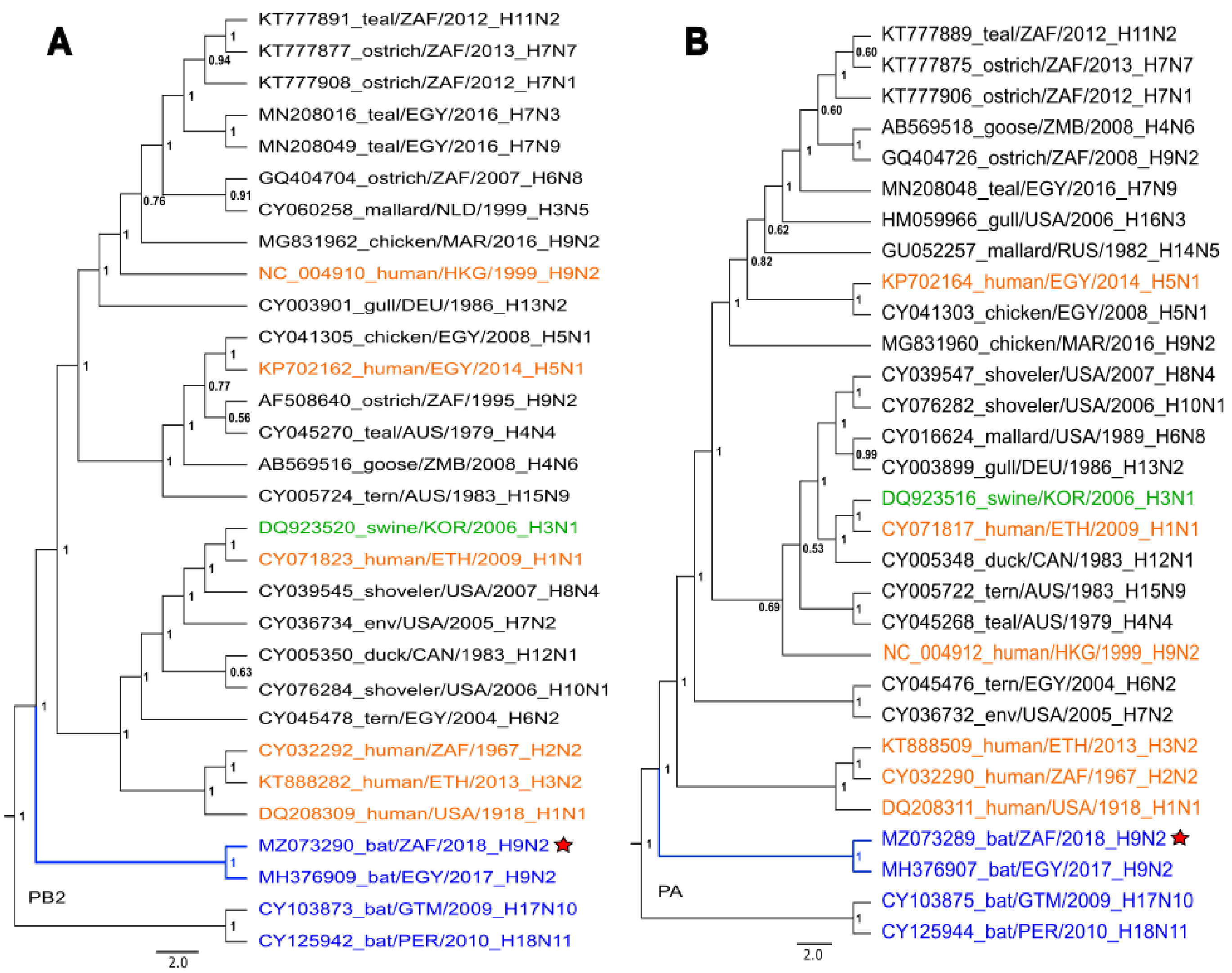

.

Figure S1: Phylogenetic trees of the polymerase gene segments where (A) represents segment 1 referred to as the PB2 gene (accession: MZ073290) and (B) represents segment 3 denoted as the PA gene (accession: MZ073289). The MCMC chain setting at 15 million samples for every 1000 states and included a burn-in of 10%. The tree's numerical values represent each node's posterior probability. All posterior values below 0.5 have been removed for presentation. The scale indicates the average number of nucleotide substitutions per site. Blue indicates the bat-borne influenza viruses, whereas orange highlights human infections, and green represents IAVs originating from swine. The red star symbol is used to mark the influenza A virus detected in South Africa from an Egyptian fruit bat colony in Limpopo.

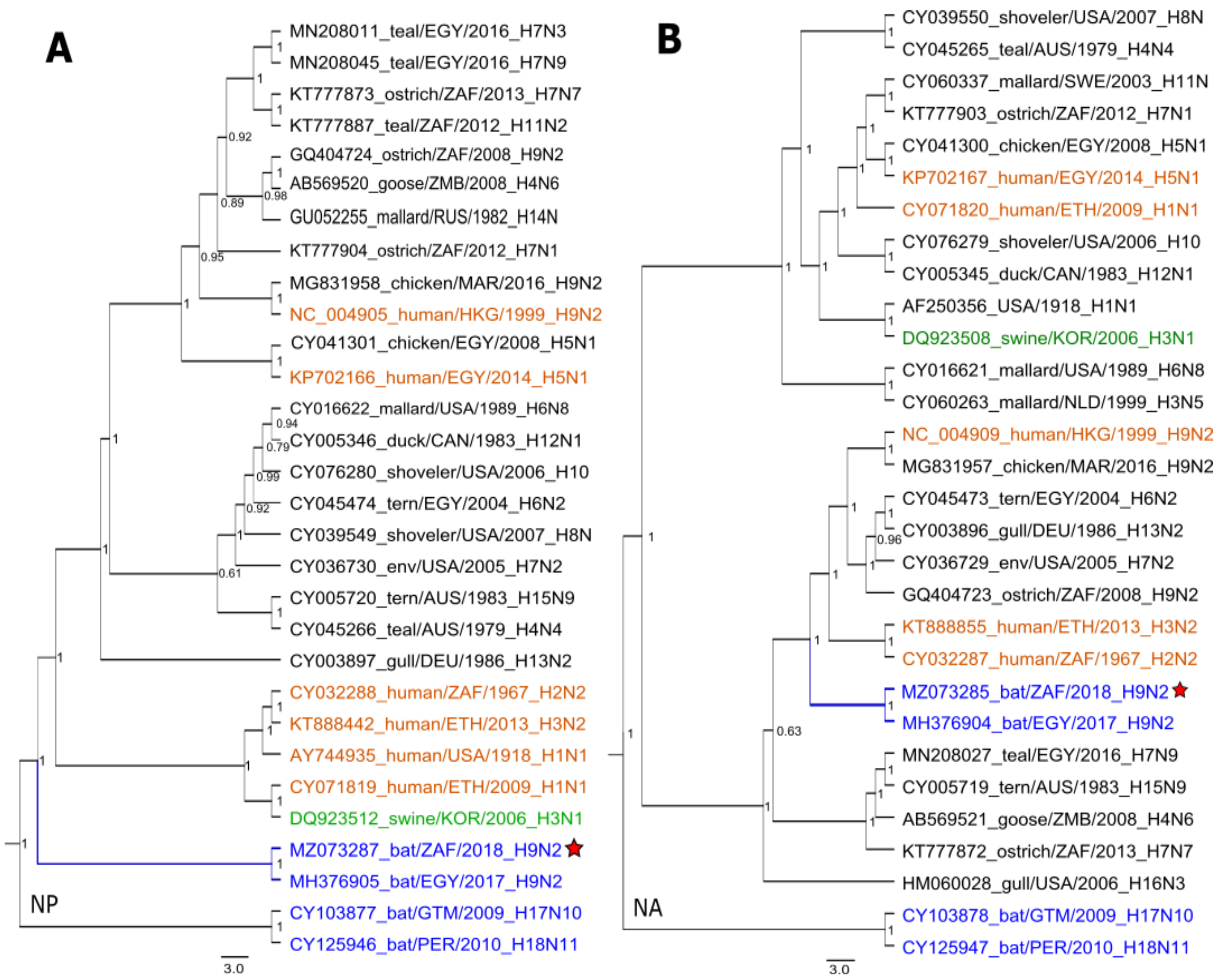

Figure S2: Phylogenetic trees for the remaining Bat/RSA/2018 (H9N2) gene-segments sequenced, where (A) represents segment 5 as the NP gene (accession: MZ073287), and (B) representing segment 6 as the NA gene (accession: MZ073285). The MCMC chain setting was set at 15 million samples for every 1000 states and included a burn-in of 10%. The tree's numerical values represent each node's posterior probability. All posterior values below 0.5 have been removed for presentation. The scale indicates the average number of nucleotide substitutions per site. Blue indicates the bat-borne influenza viruses, whereas orange highlights human infections, and green represents IAVs originating from swine. The red star symbol marks the influenza A virus detected in South Africa from an Egyptian fruit bat colony in Limpopo.

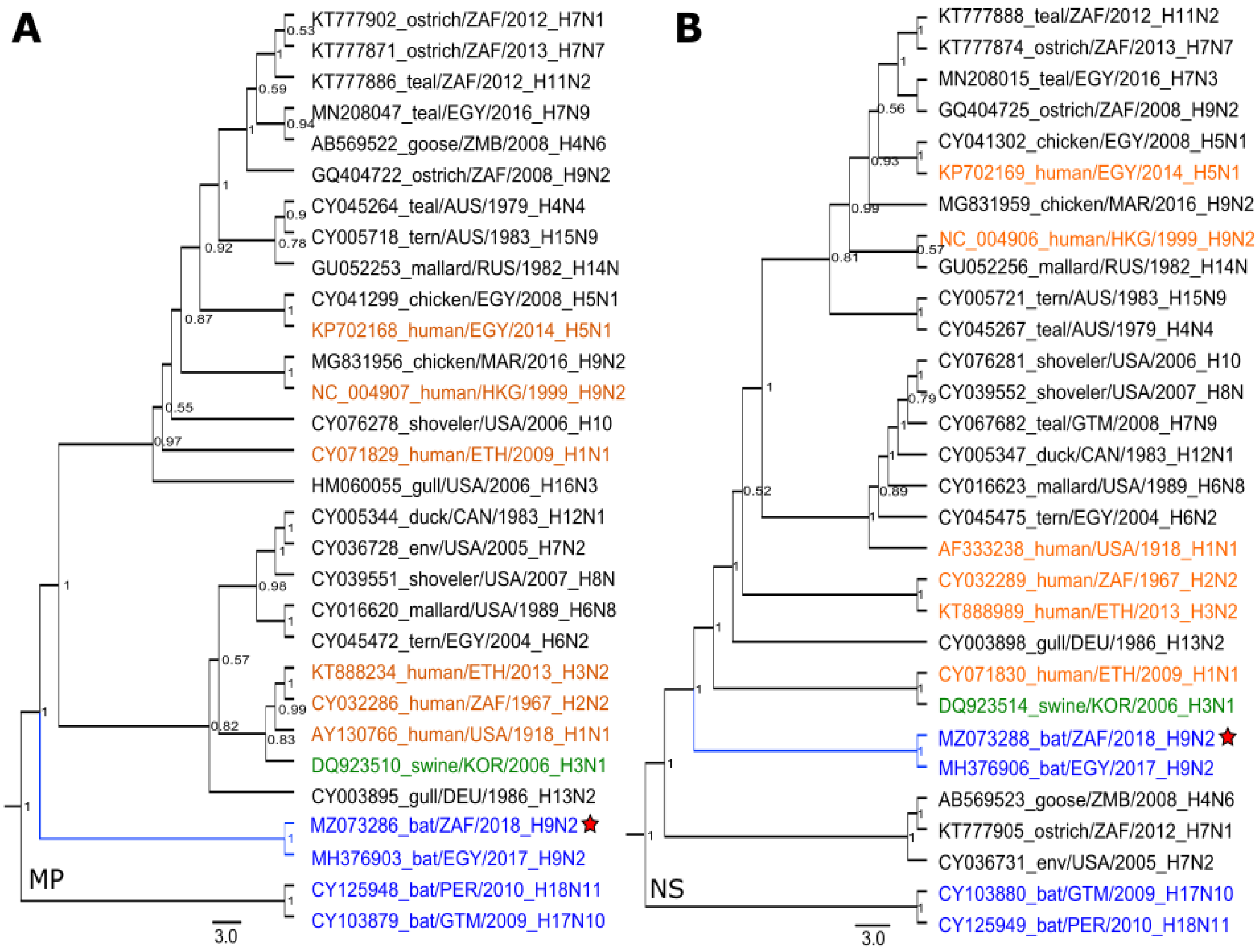

Figure S3: Phylogenetic trees for the remaining Bat/RSA/2018 (H9N2) gene-segments sequenced, where (A) represents segment 7 known as the M gene (accession: MZ073286), and (B) represents segment 8 commonly known as the NS gene segment (accession: MZ073288). The MCMC chain setting was set at 15 million samples for every 1000 states and included a burn-in of 10%. The tree's numerical values represent each node's posterior probability. All posterior values below 0.5 have been removed for presentation. The scale indicates the average number of nucleotide substitutions per site. Blue indicates the bat-borne influenza viruses, whereas orange highlights human infections, and green represents IAVs originating from swine. The red star symbol marks the influenza A virus detected in South Africa from an Egyptian fruit bat colony in Limpopo.

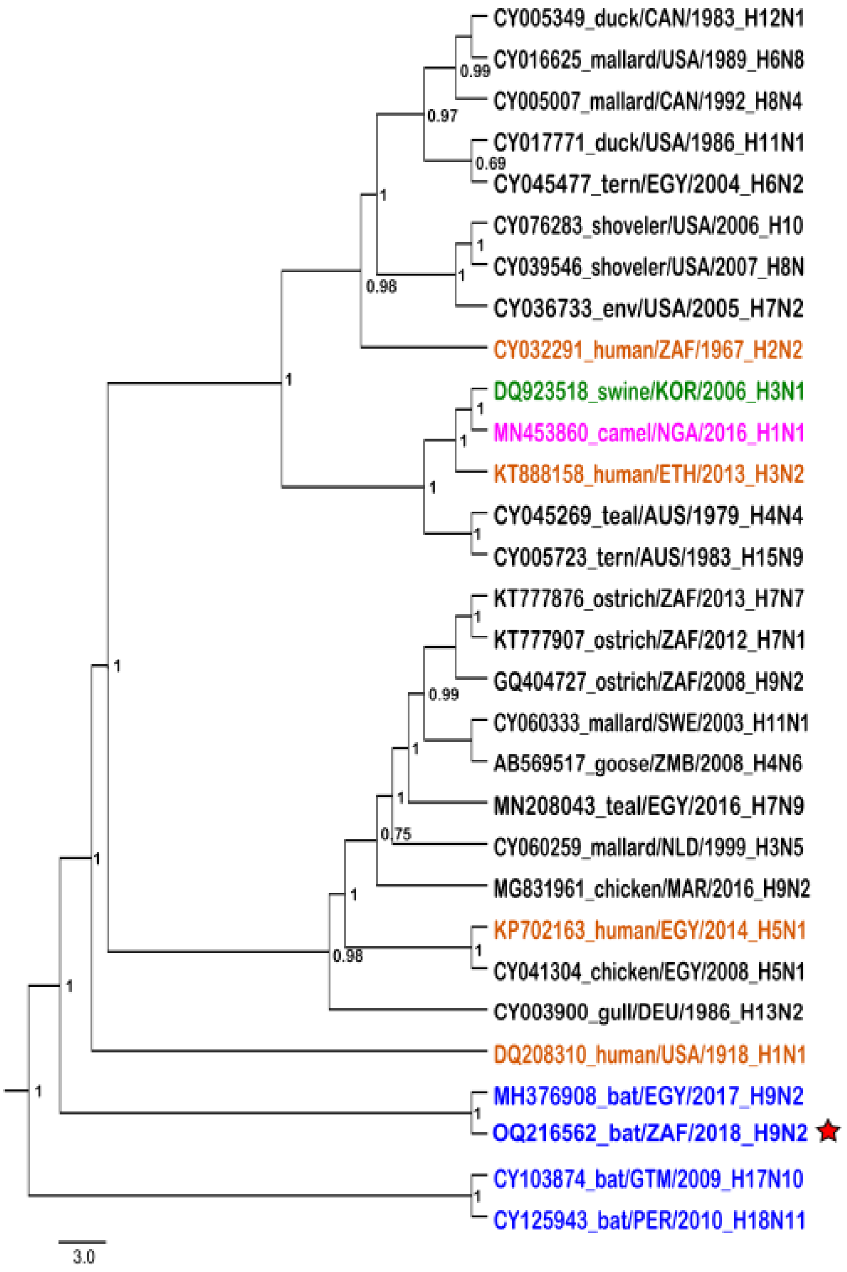

Figure S4: Phylogenetic tree for the partial PB1 fragment of the Bat/UP556/RSA/2018 (H9N2) virus (accession: OQ216562). The MCMC chain setting was set at 15 million samples for every 1000 states and included a burn-in of 10%. The tree's numerical values represent each node's posterior probability; however, all posterior values below 0.5 have been removed for presentation. The scale indicates the average number of nucleotide substitutions per site. Blue indicates the bat-borne influenza viruses, orange highlights human infections, green represents IAVs originating from swine, and purple indicates other mammalian hosts. The red star symbol marks the influenza A virus detected in South Africa from an Egyptian fruit bat colony in Limpopo.

Table S2: Important residues present within the Bat/UPE556/RSA/2018 (H9N2) segment viral proteins.

IAV segment proteins	Importance	Residue(s) present	References
**PB2**	Polymerase activity	R597, P620, F621, R646, R650	[1]
	Cap-binding	F363, F404	[2]
	PB1-unit contact	2-ERI-4, 6-EL-7	[2]
	Mitochondrial targeting signal	N9	[3]
	Minor nuclear localization signal	737-RKR-739	[2]
	Major nuclear localization signal	752-KRIR-755	[2]
	Mammalian transmission and virulence	S199, V504, N701	[4, 5]
	Association with avian influenza	K702, E627	[6]
**PA**	Endonuclease catalytic domain	E80, D108, E119	[2]
	Cap-binding	K102, H510	[2]
	PB1-unit binding	Q408, N412, P620, I621, E623, Q670, R673, W706	[2]
	Mammalian transmission and virulence	N55, Y241, S404, V127, L550, L672	[4, 7, 8]
**PA-X**	RNA endonuclease domain	P107, D108, E119, K134	[9]
	Enhanced host shutoff	P28, S65	[10]
	Increased virulence	R195K	[11]
	Protein and host shutoff	K195, K198, K202, K206	[9]
	Enhanced viral replication and host shutoff	233-252 end of the PA-X protein	[12]
**H**	Receptor binding site (identical to MH376902)	N166, H191, E197, Q198, G232, Q234, G235, R236	[4]
	Cleavage site (identical to MH376902)	333-PAIQTRGLF-341	[4]

IAV segment proteins	Importance	Residue(s) present	References
**H**	Glycosylation sites (identical to MH376902)	298-NSTL-301, 305-NISK-308	[4]
**NP**	nuclear localization signal	3-sxGTKRSYxxM-13	[13]
	Early adaptation markers for Mx antiviral resistance	K305, K351	[14]
	High pathogenicity in chickens	V105, K184	[15]
**N**	Highly conserved amino acid sequence of the cytoplasmic tail	1-MNPNQK-6	[16]
	Cysteine stabilization of protein structure	C58	[17]
	N2 catalytic resides	R118, D151, R152, R224, R276, R292, R371, Y406	[16]
	N2 structural framework amino acids	R156, W178, S179, N198, I222, E227, H274, N294, E425	[16]
	Highly conserved glycosylation signals	143-NGT, 231-NGT	[18]
	Second sialidase binding site (2SBS) 370-loop	367-SKDSRSG-373	[19, 20]
	Second sialidase binding site (2SBS) 400-loop	399-GNTDWS-404	[19, 20]
	Second sialidase binding site (2SBS) 430-loop	431-PRE-433	[19, 20]
	Second sialidase binding site (2SBS) conserved residues	S367 (linked to avian N2), S370, S372, N400, and W403	[19, 20]
**M1**	Nuclear localization signal	101- RKLKR-105	[21]
	Viral replication	76-RRR-78	[22]
**M2**	Conserved cysteine residues to stabilize the M2-tetramer	C17, C19	[23]
	Increased virulence	P69	[24]

IAV segment proteins	Importance	Residue(s) present	References
**NS1**	Nuclear localization signal	36-DRLKR-40	[25]
	CPSF30-binding	F103, M106	[26]
	PKR-binding	I123, M124, K126, N1127	[27]
	Nuclear export signal sequence	138- FNKLETLVLL-147	[28]
**NEP**	Nuclear export signal	12-ILXRMSKMQL-21	[29]

Table S3: Accession numbers and relevant details of the influenza A virus sequences used for primer design, phylogenetic analysis, and protein sequence comparison

Influenza A virus name classification in GenBank	Host	Species name	Country code	Year	Sub-type	Accession number (GenBank)							
						PB2	PB1	PA	NP	NA	M	NS	HA
A/California/04/2009 (H1N1)	Human	*Homo sapiens*	USA	2009	H1N1	FJ969516	GQ377049	FJ969515	FJ969512	FJ969517	FJ969513	FJ969514	GQ117044
A/California/7/2009 (H1N1)	Human	*Homo sapiens*	USA	2009	H1N1	MN596844	MN596850	MN596848	MN596846	MN596847	MN596849	MN596843	MN596845
A/Malaysia/1954 (H1N1)	Human	*Homo sapiens*	MYS	1954	H1N1	CY009347	CY009346	CY009345	CY009343	CY009342	CY009341	CY009344	CY009340
A/Brazil/11/1978 (H1N1)	Human	*Homo sapiens*	BRA	1978	H1N1	CY020300	CY033583	CY020298	CY020296	CY020295	CY020294	CY020297	CY020293
A/swine/Tennessee/1/1975 (H1N1)	Swine	*n.s (Sus sp.)*	USA	1975	H1N1	CY022404	CY022403	CY022402	CY022400	CY022399	CY022398	CY022401	CY022397
A/Puerto Rico/8/1934 (H1N1)	Human	*Homo sapiens*	PRI	1934	H1N1	CY033584	CY033583	CY033582	CY033580	CY033579	CY033578	CY033581	CY033577
A/Camelus dromedarius/Nigeria/NV1337/2016 (H1N1)	Camel	*Camelus dromedarius*	NGA	2016	H1N1	MN453859	MN453860	MN453861	MN453863	MN453864	MN453865	MN453866	MN453862
A/swine/Hong Kong/103/1993 (H1N1)	Swine	*n.s (Sus sp.)*	HKG	1993	H1N1	GQ229281	GQ229276	GQ229280	GQ229278	GQ229282	GQ229279	GQ229275	GQ229277
A/red-winged tinamou/Argentina/MP1/2008 (H1N1)	Avian	*Rhynchotus rufescens*	ARG	2008	H1N1	GQ369462	GQ385248	GQ379899	GQ168616	GQ143810	GQ223719	GQ202688	GQ168615
A/Brevig Mission/1/1918 (H1N1)	Human	*Homo sapiens*	USA	1918	H1N1	DQ208309	DQ208310	DQ208311	AY744935	AF250356	AY130766	AF333238	AF116575
A/common teal/Netherlands/10/2000 (H1N1)	Avian	*Anas crecca*	NLD	2000	H1N1	CY060175	CY060176	CY060177	CY060179	CY060180	CY060181	CY060182	CY060178
A/Wisconsin/10/98 (H1N1)	Human	*Homo sapiens*	USA	1998	H1N1	AF342824	AF342823	AF342822	AF342819	AF342820	AF342818	AF342817	AF342821
A/swine/Argentina/CIP051-StaFeN2/2010 (H1N2)	Swine	*n.s (Sus sp.)*	ARG	2010	H1N2	CY075861	CY075862	CY075863	CY075865	CY075866	CY075867	CY075868	CY075864
A/swine/Zhejiang/1/2004 (H1N2)	Swine	*n.s (Sus sp.)*	CHN	2004	H1N2	DQ139325	DQ139327	DQ139326	DQ139324	DQ139321	DQ139322	DQ139323	DQ139320
A/mallard/Alberta/42/1977 (H1N6)	Avian	*Anas platyrhynchos*	CAN	1977	H1N6	CY004465	CY004464	CY004463	CY004461	CY004460	CY004459	CY004462	CY004458
A/Egyptian goose/South Africa/AI1448/2007 (H1N8)	Avian	*Alopochen aegyptiaca*	RSA	2007	H1N8	GQ404712	GQ404711	GQ404710	GQ404708	GQ404707	GQ404706	GQ404709	GQ404705
A/semi-palmated sandpiper/Brazil/43/1990 (H2N1)	Avian	*Calidris pusilla*	BRA	1990	H2N1	CY005420	CY005419	CY005418	CY005416	CY005415	CY005414	CY005417	CY005413
A/Johannesburg/617/1967 (H2N2)	Human	*Homo sapiens*	RSA	1967	H2N2	CY032292	CY032291	CY032290	CY032288	CY032287	CY032286	CY032289	CY032285
A/El Salvador/2/1957 (H2N2)	Human	*Homo sapiens*	SLV	1957	H2N2	HM204774	HM204773	HM204772	HM204770	HM204769	HM204768	HM204771	HM204767
A/duck/Nanchang/4-184/2000 (H2N9)	Avian	*n.s (Anas sp.)*	CHN	2000	H2N9	CY005444	CY005443	CY005442	CY005440	CY005439	CY005438	CY005441	CY014609
A/swine/Korea/PZ72-1/2006 (H3N1)	Swine	*n.s (Sus sp.)*	KOR	2006	H3N1	DQ923520	DQ923518	DQ923516	DQ923512	DQ923508	DQ923510	DQ923514	DQ923506

Influenza A virus name classification in GenBank	Host	Species name	Country code	Year	Sub-type	Accession number (GenBank)							
						PB2	PB1	PA	NP	NA	M	NS	HA
A/mallard duck/ALB/26/1976 (H3N1)	Avian	*Anas platyrhynchos*	CAN	1976	H3N1	CY004716	CY004715	CY004714	CY004712	CY004711	CY004710	CY004713	CY005943
A/blue-winged teal/Guatemala/CIP049H108-67/2012 (H3N2)	Avian	*Spatula discors*	GTM	2012	H3N2					KX960444			
A/Addis Ababa/1514A07305892N/2013 (H3N2)	Human	*Homo sapiens*	ETH	2013	H3N2	KT888282	KT888158	KT888509	KT888442	KT888855	KT888234	KT888989	KT889256
A/Managua/410.02/2010 (H3N2)	Human	*Homo sapiens*	NIC	2010	H3N2	CY074842	CY074841	CY074840	CY074838	CY074837	CY074836	CY074839	CY074835
A/New York/802/1993 (H3N2)	Human	*Homo sapiens*	USA	1993	H3N2	CY013788	CY013787	CY013786	CY013784	CY013783	CY013782	CY013785	CY013781
A/Panama/2007/1999 (H3N2)	Human	*Homo sapiens*	PAN	1999	H3N2	DQ508862	DQ508863	DQ508864	DQ508866	DQ508867	DQ508868	DQ508869	DQ508865
A/mallard/Netherlands/2/1999 (H3N5)	Avian	*Anas platyrhynchos*	NLD	1999	H3N5	CY060258	CY060259	CY060260	CY060262	CY060263	CY060264	CY060265	CY060261
A/blue-winged teal/Guatemala/CIP049H112-60/2012 (H3N8)	Avian	*Spatula discors*	GTM	2012	H3N8				KY644505				
A/equine/Johannesburg/1/1986 (H3N8)	Equine	*n.s (Equus sp.)*	RSA	1986	H3N8	CY032960	CY032959	CY032958	CY032956	CY032955	CY032954	CY032957	CY032953
A/equine/Sao Paulo/6/1963 (H3N8)	Equine	*n.s (Equus sp.)*	BRA	1963	H3N8	CY032300	CY032299	CY032298	CY032296	CY032295	CY032294	CY032297	CY032293
A/black duck/Perth/699/1978 (H3N8)	Avian	*Anas rubripes*	AUS	1978	H3N8	CY028643	CY028642	CY028641	CY028639	CY028638	CY028637	CY028640	CY028636
A/mallard/Alberta/47/98 (H4N1)	Avian	*Anas platyrhynchos*	CAN	1998	H4N1	CY004932	CY004931	CY004930	CY004928	CY004927	CY004926	CY004929	CY004925
A/gray teal/Western Australia/1840/1979 (H4N4)	Avian	*Anas gracilis*	AUS	1979	H4N4	CY045270	CY045269	CY045268	CY045266	CY045265	CY045264	CY045267	CY045263
A/goose/Zambia/07/2008 (H4N6)	Avian	*n.s (Anser sp.)*	ZMB	2008	H4N6	AB569516	AB569517	AB569518	AB569520	AB569521	AB569522	AB569523	AB569519
A/mallard/Manitoba/23912/2007 (H4N7)	Avian	*Anas platyrhynchos*	CAN	2007	H4N7	CY047695	CY047694	CY047693	CY047691	CY047690	CY047689	CY047692	CY047688
A/slaty-backed gull/Japan/6KS0191/2006 (H4N8)	Avian	*Larus schistisagus*	JPN	2006	H4N8	CY080238	CY080237	CY080236	CY080234	CY080233	CY080232	CY080235	CY080231
A/Egypt/MOH-NRC-7271/2014 (H5N1)	Human	*Homo sapiens*	EGY	2014	H5N1	KP702162	KP702163	KP702164	KP702166	KP702167	KP702168	KP702169	KP702165
A/chicken/Egypt/0891/2008 (H5N1)	Avian	*n.s (Gallus sp.)*	EGY	2008	H5N1	CY041305	CY041304	CY041303	CY041301	CY041300	CY041299	CY041302	CY041298
A/Viet Nam/1203/2004 (H5N1)	Human	*Homo sapiens*	VNM	2004	H5N1	AY818126	AY818129	AY818132	AY818138	AY818141	AY818144	AY818147	AY818135
A/turkey/England/50-92/1991 (H5N1)	Avian	*n.s (Meleagris sp.)*	GBR	1991	H5N1	EU636689	EU636690	EU636691	EU636693	EU636694	EU636695	EU636696	EU636692
A/chicken/Chis/15224/1997 (H5N2)	Avian	*n.s (Gallus sp.)*	MEX	1997	H5N2	CY005844	CY006041	CY005843	CY005841	CY005840	CY005839	CY005842	CY014717
A/duck/New Zealand/41/1984 (H5N2)	Avian	*n.s (Anas sp.)*	NZL	1984	H5N2	CY005764	CY005763	CY005762	CY005760	CY014641	CY005759	CY005761	CY014640
A/mallard duck/ALB/57/1976 (H5N2)	Avian	*Anas platyrhynchos*	CAN	1976	H5N2	CY004324	CY004323	CY004322	CY004320	CY004319	CY004318	CY004321	CY005918
A/northern pintail/Alaska/16-041335-7/2016 (H5N2)	Avian	*Spatula clypeata*	USA	2016	H5N2	MH546896	MH546897	MH546898	MH546900	MH546901	MH546902	MH546903	MH546163

Influenza A virus name classification in GenBank	Host	Species name	Country code	Year	Sub-type	Accession number (GenBank)							
						PB2	PB1	PA	NP	NA	M	NS	HA
A/gray teal/Australia/1/1979 (H6N1)	Avian	*Anas gracilis*	AUS	1979	H6N1	CY005671	CY005670	CY005669	CY005667	CY014624	CY005666	CY005668	CY014623
A/chicken/South Africa/AL19/02 (H6N2)	Avian	*n.s (Gallus sp.)*	RSA	2002	H6N2	DQ408516	DQ408515	DQ408514	DQ408512	DQ408511	DQ408510	DQ408513	DQ408509
A/rosy-billed pochard/Argentina/CIP051-557/2007 (H6N2)	Avian	*Netta peposaca*	ARG	2007	H6N2	CY067691	CY067692	CY067693	CY067695	CY067696	CY067697	CY067698	CY067694
A/whiskered tern/Egypt/04/2004 (H6N2)	Avian	*Chlidonias hybrida*	EGY	2004	H6N2	CY045478	CY045477	CY045476	CY045474	CY045473	CY045472	CY045475	CY045471
A/duck/Beijing/TZ/2014 (H6N2)	Avian	*n.s (Anas sp.)*	CHN	2014	H6N2	KX518658	KX518659	KX518660	KX518662	KX518663	KX518664	KX518665	KX518661
A/ostrich/South Africa/AI1447/2007 (H6N8)	Avian	*Struthio camelus*	RSA	2007	H6N8	GQ404704	GQ404703	GQ404702	GQ404700	GQ404699	GQ404698	GQ404701	GQ404697
A/Bewick's swan/Netherlands/2/2005 (H6N8)	Avian	*Cygnus columbianus*	NDL	2005	H6N8	DQ822195	DQ822196	DQ822197	DQ822191	DQ822192	DQ822193	DQ822194	DQ822190
A/mallard/Ohio/64/1989 (H6N8)	Avian	*Anas platyrhynchos*	USA	1989	H6N8	CY016626	CY016625	CY016624	CY016622	CY016621	CY016620	CY016623	CY016619
A/ostrich/South Africa/ORD/2012 (H7N1)	Avian	*Struthio camelus*	RSA	2012	H7N1	KT777908	KT777907	KT777906	KT777904	KT777903	KT777902	KT777905	KT777901
A/common pochard/XiangHai/420/2010 (H7N1)	Avian	*Aythya ferina*	CHN	2010	H7N1							KU663409	
A/duck/Mongolia/47/2001 (H7N1)	Avian	*n.s (Anas sp.)*	MNG	2001	H7N1	AB473548	AB268552	AB268553	AB268554	AB302788	AB268555	AB268556	AB268557
A/chicken/Rostock/45/1934 (H7N1)	Avian	*n.s (Gallus sp.)*	DEU	1934	H7N1	CY077417	CY077418	CY077419	CY077421	CY077422	CY077423	CY077424	CY077420
A/environment/New York/30732-1/2005 (H7N2)	Indet	Indet	USA	2005	H7N2	CY036734	CY036733	CY036732	CY036730	CY036729	CY036728	CY036731	CY036727
A/laughing gull/NY/2455/2000 (H7N3)	Avian	*Leucophaeus atricilla*	USA	2000	H7N3						DQ021689		
A/teal/Egypt/MB-D-487OP/2016 (H7N3)	Avian	*n.s (Anas sp.)*	EGY	2016	H7N3	MN208016						MN208015	
A/ostrich/South Africa/KRB/2013 (H7N7)	Avian	*Struthio camelus*	RSA	2013	H7N7	KT777877	KT777876	KT777875	KT777873	KT777872	KT777871	KT777874	KT777870
A/equine/Argentina/1/1977 (H7N7)	Equine	*n.s (Equus sp.)*	ARG	1977	H7N7	CY036902	CY036901	CY036900	CY036898	CY036897	CY036896	CY036899	CY036895
A/equine/Prague/1/1956 (H7N7)	Equine	*n.s (Equus sp.)*	CZE	1956	H7N7	M73519	CY096913	CY096912	CY005803	CY005802	CY005801	CY005804	X62552
A/equine/Lexington/1/1966 (H7N7)	Equine	*n.s (Equus sp.)*	USA	1966	H7N7			CY039396					
A/teal/Egypt/MB-D-621C/2016 (H7N9)	Avian	*n.s (Anas sp.)*	EGY	2016	H7N9	MN208049	MN208043	MN208048	MN208045	MN208027	MN208047	MN208046	MN208044
A/duck/Hunan/S11682/2015 (H7N9)	Avian	*n.s (Anas sp.)*	CHN	2015	H7N9	MF630450							
A/blue-winged teal/Guatemala/CIP049-02/2008 (H7N9)	Avian	*Spatula discors*	GTM	2008	H7N9	CY067675	CY067676	CY067677	CY067679	CY067680	CY067681	CY067682	CY067678
A/chicken/Guangdong/SD014/2014 (H7N9)	Avian	*n.s (Gallus sp.)*	CHN	2014	H7N9	MN037513	MN037514	MN037515	MN037517	MN037518	MN037519	MN037520	MN037516
A/chicken/China/ZSM/2017 (H7N9)	Avian	*n.s (Gallus sp.)*	CHN	2017	H7N9	MH553137	MH553138	MH553139	MH553141	MH553142	MH553143	MH553144	MH553140

Influenza A virus name classification in GenBank	Host	Species name	Country code	Year	Sub-type	Accession number (GenBank)							
						PB2	PB1	PA	NP	NA	M	NS	HA
A/mallard/Alberta/194/1992 (H8N4)	Avian	*Anas platyrhynchos*	CAN	1992	H8N4	CY005008	CY005007	CY005006	CY005004	CY005003	CY005002	CY005005	CY005972
A/turkey/Ontario/6118/1968 (H8N4)	Avian	*n.s (Meleagris sp.)*	CAN	1968	H8N4	CY005831	CY014662	CY005830	CY005829	CY014660	CY005828	CY014661	CY014659
A/northern shoveler/California/AKS273/2007 (H8N4)	Avian	*Spatula clypeata*	USA	2007	H8N4	CY039545	CY039546	CY039547	CY039549	CY039550	CY039551	CY039552	CY039548
A/Bat/Egypt/381OP/2017 (H9N2)	Bat	*Rousettus aegyptiacus*	EGY	2017	H9N2	MH376909	MH376908	MH376907	MH376905	MH376904	MH376903	MH376906	MH376902
A/Rousettus aegyptiacus/South Africa/UPE556/2018 (H9N2)	Bat	*Rousettus aegyptiacus*	RSA	2018	H9N2	MZ073290	OQ216562	MZ073289	MZ073287	MZ073285	MZ073286	MZ073288	OQ216561
A/chicken/Egypt/SCU8/2014 (H9N2)	Avian	*n.s (Gallus sp.)*	EGY	2014	H9N2	KP027612	KP027613	KP027614	KP027616	KP027617	KP027618	KP027615	KP027619
A/duck/Egypt/C9787/2014 (H9N2)	Avian	*n.s (Anas sp.)*	EGY	2014	H9N2	KX000804	KX000841	KX000755	KX000767	KX000738	KX000728	KX000747	KX000751
A/chicken/JinShui/JS1002/2018 (H9N2)	Avian	*n.s (Gallus sp.)*	CHN	2018	H9N2	MH375882	MH375881	MH375880	MH375875	MH375878	MH375877	MH375879	MH375876
A/Hong Kong/1073/99 (H9N2)	Human	*Homo sapiens*	HKG	1999	H9N2	NC_004910	NC_004911	NC_004912	NC_004905	NC_004909	NC_004907	NC_004906	NC_004908
A/chicken/Iraq/4/2016 (H9N2)	Avian	*n.s (Gallus sp.)*	IRQ	2016	H9N2	MH879796	MH879797	MH879798	MH879800	MH879801	MH879802	MH879803	MH879799
A/broiler chicken/Casablanca/16VIR9564-1/2016 (H9N2)	Avian	*n.s (Gallus sp.)*	MAR	2016	H9N2	MG831962	MG831961	MG831960	MG831958	MG831957	MG831956	MG831959	MG831955
A/chicken/Yokohama/aq55/2001 (H9N2)	Avian	*n.s (Gallus sp.)*	JPN	2001	H9N2	AB256671	AB256672	AB256673	AB256675	AB256676	AB256677	AB256678	AB256674
A/Ostrich/South Africa/9508103/95 (H9N2)	Avian	*Struthio camelus*	RSA	1995	H9N2	AF508640	AF508618	AF508662	AF508596	AF508575	AF508684	AF508705	AF508554
A/mallard/Alberta/11/1991 (H9N2)	Avian	*Anas platyrhynchos*	CAN	1991	H9N2	CY005153	CY005152	CY005151	CY005149	CY014591	CY005148	CY005150	CY005990
A/quail/Bangladesh/28122/2016 (H9N2)	Avian	*n.s (Coturnix sp.)*	BGD	2016	H9N2	MN037521	MN037522	MN037523	MN037525	MN037526	MN037527	MN037528	MN037524
A/ostrich/South Africa/AI1586/2008 (H9N2)	Avian	*Struthio camelus*	RSA	2008	H9N2	-	GQ404727	GQ404726	GQ404724	GQ404723	GQ404722	GQ404725	GQ404721
A/ruddy turnstone/DE/773/1988 (H9N6)	Avian	*Arenaria interpres*	DEU	1988	H9N6	CY004574	CY004573	CY004572	CY004570	CY004569	CY004568	CY004571	CY005934
A/duck/Hong Kong/147/1977 (H9N6)	Avian	*n.s (Anas sp.)*	HKG	1977	H9N6	CY005646	CY005645	CY005644	CY005642	CY005641	CY005640	CY005643	CY005639
A/northern shoveler/Washington/44249-700/2006 (H10N1)	Avian	*Spatula clypeata*	USA	2006	H10N1	CY076284	CY076283	CY076282	CY076280	CY076279	CY076278	CY076281	CY076277
A/mallard/ALB/5/1995 (H10N1)	Avian	*Anas platyrhynchos*	CAN	1995	H10N1	CY005231	CY005230	CY005229	CY005227	CY005226	CY005225	CY005228	CY005997
A/quail/Italy/1117/1965 (H10N8)	Avian	*n.s (Coturnix sp.)*	ITA	1965	H10N8	CY005800	CY005799	CY014645	CY005797	CY005796	CY005795	CY005798	CY014644
A/Black Duck/Ohio/194/1986 (H11N1)	Avian	*Anas rubripes*	USA	1986	H11N1	CY017772	CY017771	CY017770	CY017768	CY017767	CY017766	CY017769	CY017765
A/mallard/Sweden/58/2003 (H11N1)	Avian	*Anas platyrhynchos*	SWE	2003	H11N1	CY060332	CY060333	CY060334	CY060336	CY060337	CY060338	CY060339	CY060335
A/aquatic bird/India/NIV-17095/2007 (H11N1)	Avian	*Platalea leucorodia*	IND	2007	H11N1	CY055172	CY055173	CY055174	CY055176	CY055177	CY055178	CY055179	CY055175

Influenza A virus name classification in GenBank	Host	Species name	Country code	Year	Sub-type	Accession number (GenBank)							
						PB2	PB1	PA	NP	NA	M	NS	HA
A/mallard duck/Alberta/342/1983 (H12N1)	Avian	*Anas platyrhynchos*	CAN	1983	H12N1	CY005350	CY005349	CY005348	CY005346	CY005345	CY005344	CY005347	AF310991
A/bar headed goose/Mongolia/143/2005 (H12N3)	Avian	*Anser indicus*	MNG	2005	H12N3	GQ907293	GQ907292	GQ907291	GQ907289	GQ907288	GQ907287	GQ907290	GQ907286
A/northern shoveler/Mississippi/09OS025/2009 (H12N5)	Avian	*Spatula clypeata*	USA	2009	H12N5	CY079395	CY079394	CY079393	CY079391	CY079390	CY079389	CY079392	CY079388
A/herring gull/DE/475/1986 (H13N2)	Avian	*Larus argentatus*	DEU	1986	H13N2	CY003901	CY003900	CY003899	CY003897	CY003896	CY003895	CY003898	CY005914
A/Mongolian gull/Mongolia/401/2007 (H13N6)	Avian	*Larus mongolicus*	MNG	2007	H13N6	GQ907317	GQ907316	GQ907315	GQ907313	GQ907312	GQ907311	GQ907314	GQ907310
A/kelp gull/Argentina/LDC4/2006 (H13N9)	Avian	*Larus dominicanus*	ARG	2006	H13N9	EU523143	EU523142	EU523141	EU523140	EU523137	EU523138	EU523139	EU523136
A/mallard/Astrakhan/263/1982 (H14N5)	Avian	*Anas platyrhynchos*	RUS	1982	H14N5	GU052259	GU052258	GU052257	GU052255	GU052254	GU052253	GU052256	M35997
A/sooty tern/Western Australia/2190/1983 (H15N9)	Avian	*Onychoprion fuscatus*	AUS	1983	H15N9	CY005724	CY005723	CY005722	CY005720	CY005719	CY005718	CY005721	CY006033
A/herring gull/Delaware Bay/712/1988 (H16N3)	Avian	*Larus smithsonianus*	USA	1988	H16N3	CY004567	CY004566	CY004565	CY004563	CY014569	CY004562	CY004564	CY005933
A/glaucous gull/Alaska/44198-027/2006 (H16N3)	Avian	*Larus hyperboreus*	USA	2006	H16N3	HM059944	HM059951	HM059966	HM060019	HM060028	HM060055	HM060065	HM059998
A/little yellow-shouldered bat/Guatemala/153/2009 (H17N10)	Bat	*Sturnira lilium*	GTM	2009	H17N10	CY103873	CY103874	CY103875	CY103877	CY103878	CY103879	CY103880	CY103876
A/little yellow-shouldered bat/Guatemala/164/2009 (H17N10)	Bat	*Sturnira lilium*	GTM	2009	H17N10	CY103881	CY103882	CY103883	CY103885	CY103886	CY103887	CY103888	CY103884
A/little yellow-shouldered bat/Guatemala/060/2010 (H17N10)	Bat	*Sturnira lilium*	GTM	2010	H17N10	CY103889	CY103890	CY103891	CY103893	CY103894	CY103895	CY103896	CY103892
A/flat-faced bat/Peru/033/2010 (18N11)	Bat	*Artibeus planirostris*	PER	2010	H18N11	CY125942	CY125943	CY125944	CY125946	CY125947	CY125948	CY125949	CY125945
A/dark fruit-eating bat/Bolivia/PBV780-781/2011 (18N11)	Bat	*Artibeus obscurus*	BOL	2011	H18N11	KR077929	KR077930	KR077931	KR077933	KR077934	KR077935	KR077936	KR077932
A/Artibeus lituratus/Brazil/2301/2012 (18N11)	Bat	*Artibeus lituratus*	BRA	2012	H18N11	MH682200	MH682201	MH682202	MH682204	MH682205	MH682206	MH682207	MH682203
A/Artibeus lituratus/Brazil/2344/2012 (18N11)	Bat	*Artibeus lituratus*	BRA	2012	H18N11	MH682208	MH682209	MH682210	MH682212	MH682213	MH682214	MH682215	MH682211

Supplementary references

1. Patel, H. and A. Kukol, *Evolutionary conservation of influenza A PB2 sequences reveals potential target sites for small molecule inhibitors.* Virology, 2017. **509**: p. 112-120.
2. Boivin, S., S. Cusack, R.W. Ruigrok, and D.J. Hart, *Influenza A virus polymerase: structural insights into replication and host adaptation mechanisms.* Journal of Biological Chemistry, 2010. **285**(37): p. 28411-28417.
3. Graef, K.M., F.T. Vreede, Y.-F. Lau, A.W. McCall, S.M. Carr, K. Subbarao, and E. Fodor, *The PB2 subunit of the influenza virus RNA polymerase affects virulence by interacting with the mitochondrial antiviral signaling protein and inhibiting expression of beta interferon.* Journal of virology, 2010. **84**(17): p. 8433-8445.
4. Kandeil, A., M.R. Gomaa, M.M. Shehata, A.N. El Taweel, S.H. Mahmoud, O. Bagato, Y. Moatasim, O. Kutkat, A.S. Kayed, and P. Dawson, *Isolation and characterization of a distinct influenza A virus from Egyptian bats.* Journal of virology, 2019. **93**(2): p. e01059-18.
5. Shi, J., G. Deng, H. Kong, C. Gu, S. Ma, X. Yin, X. Zeng, P. Cui, Y. Chen, H. Yang, X. Wan, X. Wang, L. Liu, P. Chen, Y. Jiang, J. Liu, Y. Guan, Y. Suzuki, M. Li, Z. Qu, L. Guan, J. Zang, W. Gu, S. Han, Y. Song, Y. Hu, Z. Wang, L. Gu, W. Yang, L. Liang, H. Bao, G. Tian, Y. Li, C. Qiao, L. Jiang, C. Li, Z. Bu, and H. Chen, *H7N9 virulent mutants detected in chickens in China pose an increased threat to humans.* Cell Research, 2017. **27**(12): p. 1409-1421.
6. Chin, A.W.H., N.K.C. Leong, J.M. Nicholls, and L.L.M. Poon, *Characterization of influenza A viruses with polymorphism in PB2 residues 701 and 702.* Scientific Reports, 2017. **7**(1): p. 11361.
7. Rolling, T., I. Koerner, P. Zimmermann, K. Holz, O. Haller, P. Staeheli, and G. Kochs, *Adaptive Mutations Resulting in Enhanced Polymerase Activity Contribute to High Virulence of Influenza A Virus in Mice.* Journal of Virology, 2009. **83**(13): p. 6673.
8. Samir, A., A. Adel, A. Arafa, H. Sultan, and H.A. Hussein Ahmed, *Molecular pathogenic and host range determinants of reassortant Egyptian low pathogenic avian influenza H9N2 viruses from backyard chicken.* International Journal of Veterinary Science and Medicine, 2019. **7**(1): p. 10-19.
9. Hu, J., C. Ma, and X. Liu, *PA-X: a key regulator of influenza A virus pathogenicity and host immune responses.* Medical Microbiology and Immunology, 2018. **207**(5): p. 255-269.
10. Oishi, K., S. Yamayoshi, and Y. Kawaoka, *Identification of Amino Acid Residues in Influenza A Virus PA-X That Contribute to Enhanced Shutoff Activity.* Frontiers in Microbiology, 2019. **10**(432).
11. Sun, Y., Z. Hu, X. Zhang, M. Chen, Z. Wang, G. Xu, Y. Bi, Q. Tong, M. Wang, H. Sun, J. Pu, M. Iqbal, and J. Liu, *An R195K Mutation in the PA-X Protein Increases the Virulence and Transmission of Influenza A Virus in Mammalian Hosts.* Journal of Virology, 2020. **94**(11): p. e01817-19.
12. Gao, H., H. Sun, J. Hu, L. Qi, J. Wang, X. Xiong, Y. Wang, Q. He, Y. Lin, W. Kong, L.-G. Seng, J. Pu, K.-C. Chang, X. Liu, J. Liu, and Y. Sun, *Twenty amino acids at the C-terminus of PA-X are associated with increased influenza A virus replication and pathogenicity.* The Journal of general virology, 2015. **96**(8): p. 2036-2049.
13. Naffakh, N., A. Tomoiu, M.-A. Rameix-Welti, and S. van der Werf, *Host restriction of avian influenza viruses at the level of the ribonucleoproteins.* Annu. Rev. Microbiol., 2008. **62**: p. 403-424.
14. Mucha, V., J. Hollý, E. Varečková, and F. Kostolanský, *Avian influenza A virus adaptation to the equine host and identification of host-specific markers.* Acta virologica, 2018. **62**(3): p. 266-276.
15. Tada, T., K. Suzuki, Y. Sakurai, M. Kubo, H. Okada, T. Itoh, and K. Tsukamoto, *NP Body Domain and PB2 Contribute to Increased Virulence of H5N1 Highly Pathogenic Avian Influenza Viruses in Chickens.* Journal of Virology, 2011. **85**(4): p. 1834.
16. McAuley, J.L., B.P. Gilbertson, S. Trifkovic, L.E. Brown, and J.L. McKimm-Breschkin, *Influenza Virus Neuraminidase Structure and Functions.* Frontiers in Microbiology, 2019. **10**(39).
17. Blok, J. and G.M. Air, *Variation in the membrane-insertion and" stalk" sequences in eight subtypes of influenza type A virus neuraminidase.* Biochemistry, 1982. **21**(17): p. 4001-4007.

18. Chen, W., Y. Zhong, Y. Qin, S. Sun, and Z. Li, *The Evolutionary Pattern of Glycosylation Sites in Influenza Virus (H5N1) Hemagglutinin and Neuraminidase.* PLOS ONE, 2012. **7**(11): p. e49224.
19. Du, W., M. Dai, Z. Li, G.-J. Boons, B. Peeters, F.J.M. van Kuppeveld, E. de Vries, and C.A.M. de Haan, *Substrate Binding by the Second Sialic Acid-Binding Site of Influenza A Virus N1 Neuraminidase Contributes to Enzymatic Activity.* Journal of Virology, 2018. **92**(20): p. e01243-18.
20. Varghese, J.N., P.M. Colman, A. van Donkelaar, T.J. Blick, A. Sahasrabudhe, and J.L. McKimm-Breschkin, *Structural evidence for a second sialic acid binding site in avian influenza virus neuraminidases.* Proceedings of the National Academy of Sciences, 1997. **94**(22): p. 11808-11812.
21. Ye, Z., D. Robinson, and R.R. Wagner, *Nucleus-targeting domain of the matrix protein (M1) of influenza virus.* Journal of Virology, 1995. **69**(3): p. 1964-1970.
22. Das, S.C., S. Watanabe, M. Hatta, T. Noda, G. Neumann, M. Ozawa, and Y. Kawaoka, *The Highly Conserved Arginine Residues at Positions 76 through 78 of Influenza A Virus Matrix Protein M1 Play an Important Role in Viral Replication by Affecting the Intracellular Localization of M1.* Journal of Virology, 2012. **86**(3): p. 1522.
23. Holsinger, L.J. and R. Alams, *Influenza virus M2 integral membrane protein is a homotetramer stabilized by formation of disulfide bonds.* Virology, 1991. **183**(1): p. 32-43.
24. Hassanin, K.M.A. and A.S. Abdel-Moneim, *Evolution of an avian H5N1 influenza A virus escape mutant.* World journal of virology, 2013. **2**(4): p. 160-169.
25. Melén, K., L. Kinnunen, R. Fagerlund, N. Ikonen, K.Y. Twu, R.M. Krug, and I. Julkunen, *Nuclear and Nucleolar Targeting of Influenza A Virus NS1 Protein: Striking Differences between Different Virus Subtypes.* Journal of Virology, 2007. **81**(11): p. 5995.
26. Kuo, R.-L. and R.M. Krug, *Influenza a virus polymerase is an integral component of the CPSF30-NS1A protein complex in infected cells.* Journal of virology, 2009. **83**(4): p. 1611-1616.
27. Min, J.-Y., S. Li, G.C. Sen, and R.M. Krug, *A site on the influenza A virus NS1 protein mediates both inhibition of PKR activation and temporal regulation of viral RNA synthesis.* Virology, 2007. **363**(1): p. 236-243.
28. Li, Y., Y. Yamakita, and R.M. Krug, *Regulation of a nuclear export signal by an adjacent inhibitory sequence: the effector domain of the influenza virus NS1 protein.* Proceedings of the National Academy of Sciences, 1998. **95**(9): p. 4864-4869.
29. O'Neill, R.E., J. Talon, and P. Palese, *The influenza virus NEP (NS2 protein) mediates the nuclear export of viral ribonucleoproteins.* The EMBO journal, 1998. **17**(1): p. 288-296.
